# Carotid Endarterectomy for the Treatment of Carotid Near-Occlusion With Recurrent Symptoms

**DOI:** 10.3389/fneur.2022.765795

**Published:** 2022-04-14

**Authors:** Jianbin Zhang, Jie Chen, Xiaojie Xu, Mingsheng Sun, Shu Chen, Peng Liu, Zhidong Ye

**Affiliations:** ^1^Department of Cardiovascular Surgery, China-Japan Friendship Hospital, Beijing, China; ^2^Department of Endocrinology, Beijing Jishuitan Hospital, The Fourth Clinical Medical College of Peking University, Beijing, China; ^3^Department of Vascular Surgery, Beijing Chao-Yang Hospital, Capital Medical University, Beijing, China; ^4^Department of Interventional Radiology, Affiliated People's Hospital of Inner Mongolia Medical University, Hohhot, China

**Keywords:** carotid endarterectomy (CEA), carotid near-occlusion (CNO), full collapse, stroke, carotid artery stenosis

## Abstract

**Objective:**

Report our preliminary experience of carotid endarterectomy (CEA) for the treatment of carotid near-occlusion (CNO) with recurrent symptoms.

**Materials and Methods:**

Retrospectively analyze the demographics, treatment detail, and outcomes data of 122 patients with CNO from 2014 to 2020. According to whether distal full collapse exists, patients were classified into the full collapse group and the non-full collapse group. The incidence of death, myocardial infarction, stroke, and other variables were compared between the two groups.

**Results:**

A total of 122 patients with CNO and recurrent symptoms were enrolled. The demographics were comparable between the two groups. Thirty-day incidence of primary endpoints was 1.85% in the full collapse group and 4.41% in the non-full collapse group. Twelve-month incidence of primary endpoints was 7.41% in the full collapse group and 4.41% in the non-full collapse group. One re-stenosis occurred in the non-full collapse group 8 months after CEA.

**Conclusion:**

For patients with CNO with recurrent symptoms, CEA is not worse than the results described in historical control groups, despite whether distal full collapse exists. The shunt is important to avoid intraoperative hypoperfusion and postoperative hyperperfusion. The long-term results should be further evaluated.

## Introduction

Carotid near-occlusion (CNO), first reported by Lippman et al. in 1970 (1), refers to a distal internal carotid artery (ICA) lumen diameter reduced beyond tight stenosis, which was previously described as pseudo-occlusion, subtotal occlusion, string sign, slim sign, functional occlusion, pre-occlusive stenosis, and critical stenosis (2). CNO is a relatively rare condition accounting for about 3% of symptomatic carotid stenosis (3) and associated with an increased risk of ipsilateral hemispheric stroke (4). CNO with or without distal full collapse is distinguished by whether the distal ICA lumen collapsed due to being thread-like. The natural history and stroke risk of CNO are not yet well-known, patients with symptomatic CNO with full collapse may be associated with a very high risk of stroke recurrence (5).

The optimal treatment strategy for CNO is still controversial. According to the 2017 Clinical Practice Guidelines of the European Society for Vascular Surgery (6), carotid endarterectomy (CEA) or carotid stenting (CAS) is not recommended in patients with symptomatic CNO. This is based on the re-analysis of the North American Symptomatic Carotid Endarterectomy Trial (NASCET) and European Carotid Surgery Trial (ECST) data, which showed that CEA is of less value for patients with CNO than for patients with severe stenosis (7, 8). However, a recent multicenter registry study showed that the risk of early recurrent stroke may be higher in medically treated patients with symptomatic CNO (9). Furthermore, more and more studies achieved good results treating CNO with CEA or CAS (10, 11). So seeking safe and effective management for those patients with CNO is urgent and worthwhile.

We have tried to treat patients with symptomatic CNO with CEA since 2014. This study aims to retrospectively report our preliminary experience of CEA for the treatment of CNO with recurrent symptoms. Then further compare the results based on whether distal full collapse exists.

## Materials and Methods

### Study Population

From January 2014 to January 2020, 444 consecutive patients with carotid artery stenosis were treated with CEA at the Cardiovascular Surgery Department of China-Japan Friendship Hospital. Among these, 122 patients with CNO with recurrent symptoms were enrolled. According to whether distal ICA full collapse exists, which was differentiated on angiography images by visual assessment, patients were divided into two groups: the full collapse group and the non-full collapse group. Then, we collected and analyzed the demographics, treatment detail, and outcomes data. The study procedures were in accordance with institutional guidelines. All data were retrospectively collected. Written informed consent was signed by all patients.

### Inclusion and Exclusion Criteria

All patients underwent digital subtraction angiography (DSA), and two or more of the following criteria should be fulfilled for the definition of CNO (7): (1) delayed cranial arrival of ICA contrast compared with the external carotid artery; (2) intracranial collaterals seen as cross-filling of contralateral vessels or ipsilateral contrast dilution; (3) obvious diameter reduction of ICA compared with contralateral ICA; and (4) ICA diameter reduction compared with ipsilateral ECA. The definition for symptoms included transient ischemia attack (TIA) and ipsilateral stroke. Stoke was defined as MRI-proved infarction and existence of clinical symptoms, with a National Institutes of Health Stroke Scale (NIHSS) score ≥1 and symptoms lasting over 24 h. Recurrent symptoms were defined as symptom onset 90 days within the previous episode of symptoms (12).

The exclusion criteria included: (1) previously received CEA or CAS; (2) no blood flow in C2 (Bouthillier classification) or distal ICA; and (3) nonatherosclerotic lesions.

### Surgical and Peri-Operative Management

Aspirin, clopidogrel, and statin were given to all the patients. Aspirin and clopidogrel were stopped 5 days before surgery and low molecular weight heparin was used instead.

Conventional CEA was carried out under general anesthesia. An incision along the anterior border of the sternocleidomastoid muscle was made. After careful dissection of subcutaneous tissue, the common, external, and ICA were isolated and controlled. Then bolus heparin (1 mg/kg) was given intravenously and the carotid artery was clamped. We cut open the common carotid artery, extended the incision to the ICA, and carefully carried out the endarterectomy. A carotid artery shunt and polytetrafluoroethylene patch were routinely used in CEA procedures. Before de-clamping the ICA, 125 ml of 20% mannitol solution and 10 mg of dexamethasone were given for the prevention of cerebral hyperperfusion syndrome (CHS).

All patients received electrocardiogram, blood pressure, and oxygen saturation monitoring for 24–48 h after surgery. Systolic blood pressure was strictly controlled to ≤140 mmHg. For patients with hyperperfusion syndrome, systolic blood pressure was controlled to ≤120 mmHg. Aspirin (100 mg/d) was started 1 day after the surgery and lasted for life, clopidogrel (75 mg/d) started 1 day after the surgery and lasted for 3 months.

### Endpoints and Follow-Up

Incidences of death, ipsilateral stroke, and myocardial infarction (MI) were defined as primary endpoints. We also collected data for other complications including cranial nerve injury and cerebral hyperperfusion syndrome (CHS). Incidence of restenosis was also recorded.

Cerebral hyperperfusion syndrome is characterized by ipsilateral headache, hypertension, seizures, and neurological deficit caused by hyperperfusion after recanalization of the carotid artery (13, 14). Restenosis was defined as ultrasound or CTA-proved >50% stenosis of the ipsilateral carotid artery (15).

Thirty-day primary endpoints and peri-operative variables were recorded. Pre- and 30-day postoperative neurological examinations were carried out. Twelve-month primary endpoints and restenosis were recorded and compared.

The endpoints were compared with historically reported results of treatment for CNO.

### Statistical Analysis

Mean ± SD was used for the presentation of continuous variables and percentages for discrete variables. A two-sided independent sample *t-*test was used for the comparison of continuous variables. For discrete variables comparison, the chi-square test or Fisher's exact test was performed. The demographics, treatment details, and outcome data were compared between the full collapse group and the non-full collapse group. SPSS version 23 (SPSS Inc., Chicago, IL, USA) was used for data analysis. A *P* value of < 0.05 was considered statistically significant.

## Results

### Demographics and Clinical Features

The final study population consists of 122 patients (104 men; mean age 67.70 ± 9.11 years), of which 54 had CNO with full collapse and 68 had CNO without full collapse. In the full collapse group, 44 were men and 10 were women and the mean age was 68.70 ± 10.16 years (range, 49–88 years). In the non-full collapse group, 60 were men and eight were women and the mean age was 66.91 ± 8.18 years (range, 43–83 years). There was no difference between groups concerning age, sex, symptoms, and risk factors for atherosclerosis. There was also no difference in the incidence of contralateral carotid artery stenosis and coronary artery disease. For patients with stroke, the NIHSS score was also similar in the two groups (Table 1).

**Table 1:** Demographics and clinical features of the study population.

	**CNO with FC**	**CNO without FC**	***P***
No.	54	68	
Age	68.70 ± 10.16	66.91 ± 8.18	0.295
Male	44	60	0.296
Hypertension	38	53	0.249
DM	24	26	0.391
Hyperlipidemia	21	25	0.810
Smoker	28	28	0.240
Carotid symptomatic			0.226
Stroke	36	38	
TIA	18	30	
NIHSS score for stroke patients	2.86 ± 1.46	3.18 ± 1.16	0.072
Contralateral lesion			0.309
Occlusion	2	4	
>70% stenosis	8	10	
50–70% stenosis	24	32	
<50% stenosis	20	22	
CAD	14	13	0.368

*CNO, carotid near-occlusion; FC, full collapse; DM, diabetes mellitus; TIA, transient ischemia attack; NIHSS, national institute of health stroke scale; modified Rankin Scale; CAD, coronary artery disease*.

Pre-operative DSA showed all patients presented with intracranial communicating artery opening. A total of 103 (84.4%) patients presented with anterior communicating artery opening, 68 (55.7%) with posterior communicating artery opening, and 18 (14.8%) with ipsilateral external-internal communicating artery opening.

### Peri-Operative Variables

The CEA technical success rate was 100%. All patients received conventional CEA with patch (Edward, USA) angioplasty. All but four patients in the full collapse group used a shunt (Aesculap, Braun, USA) during the CEA procedure. In these four patients, the diameter of the distal ICA was too thin for shunt implantation. We elevated the blood pressure to ensure distal ICA reflux pressure ≥50 mm Hg.

**Figure 1:**
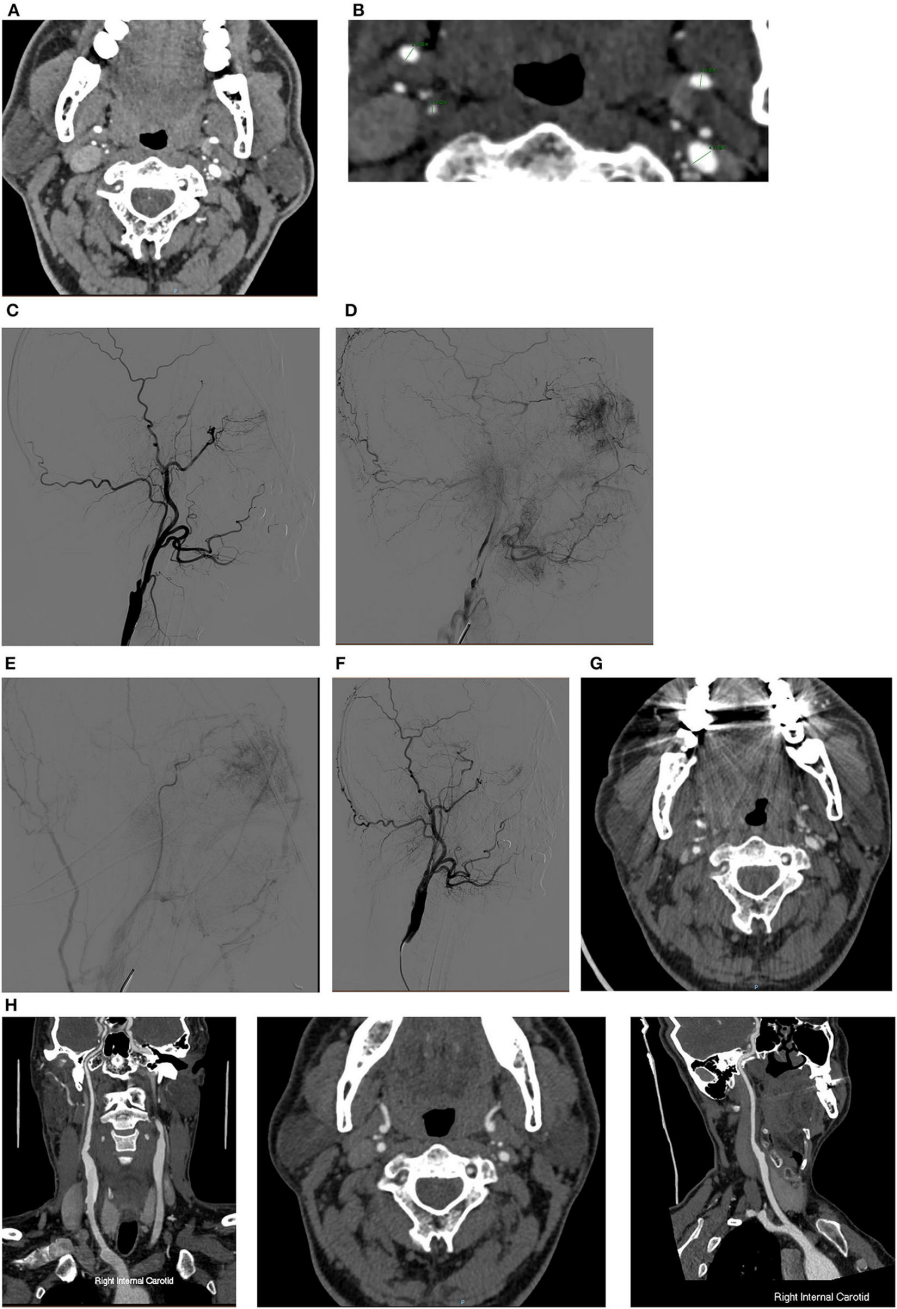
A typical case of CEA for CNO with distal full collapse. **(A,B)** CTA characteristics for CNO with distal collapse. Right ICA: 1.51 mm, right ECA: 2.86 mm, left ICA: 2.69 mm, left ECA: 4.31 mm, right ICA/left ICA: 0.56 ( ≤ 0.87), right ICA/ECA: 0.53 (≤1.27); DSA characteristics for CNO with distal collapse. **(C)** Severe stenosis of the ICA with a collapsed distal ICA; **(D)** delayed time of contrast arrival of distal ICA; **(E)** delayed antegrade flow of the patent ICA in the late arterial phase; **(F)** consistent contrast arrival of ICA and ECA after CEA. The 30-day and 12-month CTA follow-up showed the RICA is patent. **(G)** Axial and sagittal view of 30-day CTA; **(H)** axial and sagittal view of 12-month CTA.

For patients with contralateral carotid artery severe stenosis, a total of three in the full collapse group and two in the non-full collapse group received CEA and simultaneous contralateral CAS. A total of four in the full collapse group and six in the non-full collapse group received staged CAS or CEA after the current CEA procedure (Figure 1).

### Primary Endpoints and Follow-Up

The global 30-day primary endpoint rate in the study population was 3.28%. A total of six patients suffered from CHS: one presented with ophthalmodynia; two presented with postoperative delirium; and three presented with headache, of which one had severe headache, MRI showed a small amount of frontal lobe bleeding. All hyperperfusion manifestation alleviated 3–7 days after surgery.

A 30-day follow-up showed one patient in the full collapse group died because of an ipsilateral stroke. There was no difference between groups concerning primary endpoints (death, ipsilateral stroke, and MI). TIA was reported in six of the patients, two in the full collapse group and four in the non-full collapse group. The incidence of cranial nerve injury and CHS was comparable between groups. A total of one patient in the full collapse group suffered from ipsilateral asymptomatic ICA occlusion, discovered by ultrasound, and MRI showed no fresh cerebral infarction (Table 2).

**Table 2:** 30-day primary endpoints and peri-operative variables.

	**CNO with FC**	**CNO without FC**	***P***
Death	1	0	0.443
Ipsilateral stroke	1	2	0.999
Myocardial infarction	0	1	0.999
TIA	2	4	0.692
Deviation of tongue	1	1	0.999
Face numbness	3	4	0.999
Hoarseness	0	1	0.999
Hyperperfusion syndrome	2	4	0.692

*CNO, carotid near-occlusion; FC, full collapse; TIA, transient ischemia attack*.

A 12-month follow-up showed one patient in the full collapse group suffered from acute MI and sudden death. No ipsilateral stroke or TIA occurred in both the groups during the follow-up time. One restenosis occurred in the non-full collapse group 8 months after CEA and was treated with CAS (Table 3).

**Table 3:** 12-month primary endpoints and incidence of restenosis.

	**CNO with FC**	**CNO without FC**	***P***
Death	2	0	0.194
Ipsilateral stroke	1	2	0.999
Myocardial infarction	1	1	0.999
Re-stenosis	1	1	0.999

## Discussion

Since the earliest CNO cases identified from NASCET, ECST, and the Veterans Affairs trial benefited more from medical treatment than CEA. Patients with CNO were then excluded from most subsequent randomized controlled trials. So only data from cohort studies evaluating CEA, CAS, or best medical treatment (BMT) were reported (9, 16–18) and the reported results were highly heterogenous. Since the periprocedural risk for CEA decreased significantly in the past years (19), more and more investigators tried to carry out CEA for patients with CNO and achieved good results. A meta-analysis (20) showed that BMT was associated with a 4.9% 30-day stroke or death rate, compared with 2.2% for CAS and 1.8% for CEA. Heterogeneity still existed in these included studies because the inclusion criteria varied. Furthermore, most of the studies did not distinguish whether CNO was accompanied by distal full collapse or symptoms.

Indeed, whether distal full collapse or symptoms exist is a key point for patients with CNO. Though some studies showed that full collapse did not increase the risk of recurrent stroke (9), other studies showed that full collapse was associated with a poor prognosis (12). The 90-day recurrent stroke risk could reach up to 43% in patients with symptomatic CNO with full collapse and 0% for patients without full collapse (5). This result challenges the current guidelines, which suggest BMT as the first-line treatment for CNO. There are some discrepancies among the existing data. Some studies reported that BMT alone could achieve good outcomes for patients with asymptomatic CNO with full collapse (21). A recent meta-analysis (22) showed that BMT alone may not be enough to support a better prognosis than CEA or CAS for patients with CNO. Subgroup analyses showed that the proportion of CNO with full collapse was not the source of high heterogeneity. Antonopoulos et al. (23) concluded that the pooled stroke rate was 1.52% after CEA, 1.80% after CAS, and 8.39% after BMT. A downward trend over time in stroke rates after CAS and BMT could be discovered. In the NASCET study, the 30-day and 12-month ipsilateral stroke rate was 6.1 and 9.1% in no-string sign patients with CNO treated with CEA, compared with 2.3 and 18.3% in medically treated patients. In patients with CNO with string sign, the 12-month incidence of ipsilateral stroke rate was 6.7% and 11.1% for CEA and medical treatment, respectively (8). Our limited data showed that CEA could achieve acceptable results compared with the previous study, especially compared with the high ipsilateral stroke rate with medical treatment in the NASCET study. However, the current data for diagnosis, treatment, and prognosis of CNO with or without full collapse is still limited. More investigations about whether full collapse was associated with prognosis should be carried out.

In this study, the cumulative 30-day primary endpoints rate was 3.28, with 1.85% in patients with full collapse, and 4.41% in patients without distal full collapse. As for ipsilateral stroke, the incidence was 1.85% in patients with full collapse and 2.94% in patients without full collapse. The peri-operative surgical risk in patients with CNO was similar to that of severe stenosis (7). During the CEA operation, we routinely use a shunt to avoid intraoperative hypoperfusion and postoperative hyperperfusion (24). However, the distal ICA may be too thin for shunt implantation in some patients. In these situations, systolic blood pressure should be elevated to achieve better collateral perfusion compensation. Though CAS has been accepted as an alternative for CEA, a full collapse distal to CNO may limit stent expansion. Especially in lesions with severe calcification, whether concentric or eccentric, the stent is difficult to expand adequately (11). So we prefer CEA to CAS technically.

The incidence of cerebral hyperperfusion syndrome in our patient series was 4.9%. When more severe preoperative ischemia exists, a higher incidence of postoperative hyperperfusion was inferred. Moulakakis et al. (25) summarized 13 studies investigating CEA for carotid artery stenosis. The incidence of CHS was 0.4–14%, the incidence of intracranial hemorrhage was 0–0.8%. Unlike other carotid artery stenoses, pre-operative intracranial perfusion was especially poor in patients with CNO, so we should attach great importance to the prevention and treatment of CHS. Cay et al. (26) treated 59 CNO patients with CAS, 8.6% of the patients suffered from hyperperfusion syndrome during the periprocedural period. Strict blood pressure control, drugs for preventing brain edema, and oxygen-free radical scavenging drugs could decrease the incidence of CHS (27).

Several limitations may exist in this study. First, the data were collected and analyzed retrospectively. Second, the lack of cerebral perfusion imaging made it impossible for further analysis. Third, the follow-up time is relatively short, the sample size is relatively small, and it is only our preliminary investigation.

## Conclusion

In conclusion, for patients with CNO with recurrent symptoms, CEA is not worse than the results described in historical control groups, despite whether distal full collapse exists. A shunt is important to avoid intraoperative hypoperfusion and postoperative hyperperfusion. The long-term results should be further evaluated.

## Data Availability Statement

The original contributions presented in the study are included in the article/supplementary material, further inquiries can be directed to the corresponding author/s.

## Ethics Statement

The studies involving human participants were reviewed and approved by Ethics Committee of China-Japan Friendship Hospital. The patients/participants provided their written informed consent to participate in this study.

## Author Contributions

All authors listed have made a substantial, direct, and intellectual contribution to the work and approved it for publication.

## Funding

This study was supported by the Elite Medical Professionals project of China-Japan Friendship Hospital (NO. ZRJY2021-QM13).

## Conflict of Interest

The authors declare that the research was conducted in the absence of any commercial or financial relationships that could be construed as a potential conflict of interest.

## Publisher's Note

All claims expressed in this article are solely those of the authors and do not necessarily represent those of their affiliated organizations, or those of the publisher, the editors and the reviewers. Any product that may be evaluated in this article, or claim that may be made by its manufacturer, is not guaranteed or endorsed by the publisher.
